# Synbiotic Effects of the Dietary Fiber Long‐Chain Inulin and Probiotic *Lactobacillus acidophilus* W37 Can be Caused by Direct, Synergistic Stimulation of Immune Toll‐Like Receptors and Dendritic Cells

**DOI:** 10.1002/mnfr.201800251

**Published:** 2018-07-18

**Authors:** Alexia Lépine, Paul de Vos

**Affiliations:** ^1^ Immunoendocrinology Group Division of Medical Biology Department of Pathology and Medical Biology University Medical Center Groningen, University of Groningen Hanzeplein 1 9700 RB Groningen The Netherlands; ^2^ Food and Biobased Research Wageningen University and Research Bornse Weilanden 9 6708 WG Wageningen The Netherlands

**Keywords:** Caco‐2, dendritic cells, inulin‐type fructans, *Lactobacillus acidophilus* W37, *Salmonella* Typhimurium

## Abstract

**Scope:**

Synbiotic effects of dietary fibers and lactobacilli are usually explained by synergistic modulation of gut microbiota. New insight, however, has demonstrated that both dietary fibers and lactobacilli can directly stimulate immune cells and benefit consumer immunity. Here, the synergistic effects of immune active long‐chain inulin (lcITF) and *Lactobacillus acidophilus* W37 (LaW37) on dendritic cells (DCs) are investigated.

**Methods and results:**

Effects of lcITF and LaW37 alone or combined were studied on Toll‐like receptor (TLRs) signaling and cytokine secretion by DCs in the presence and absence of media of intestinal epithelial cell (IEC) exposed to the ingredients. Also, the effects of DC responses against *Salmonella* Typhimurium (STM) were investigated. Synergistic effects were observed on TLR2 and 3. Synergistic effects were not always pro‐inflammatory. LaW37 was strongly pro‐inflammatory, while cytokine responses were regulatory when combined with lcITF. Exposure of DCs to IECs medium changed the DCs’ response, which revealed synergistic enhancing effects of lcITF/LaW37 on production of IL‐6 and IL‐8. DCs’ response in the presence of STM and LaW37 were so strong that lcITF had no additional effect.

**Conclusion:**

It is demonstrated that synbiotic effects of dietary fibers and bacteria are not limited to the effects on gut microbiota but can also occur by synergistically directly stimulating IECs and/or immune cells.

## 1 Introduction

Both the consumption of dietary fibers and of beneficial bacteria such as lactobacilli have been associated with health benefits, including lowering symptoms of inflammatory diseases such as inflammatory bowel disease,^1^, ^2^, ^3^ and infections with enteropathogens such as *Salmonella* Typhimurium (STM).^4^ Despite those widely accepted beneficial effects of these food ingredients, the mechanisms behind these health effects are still not fully elucidated. It has been shown that the consumption of dietary fibers and lactobacilli may lower circulating cytokines,^5^, ^6^, ^7^, ^8^, ^9^ and that some combinations of fibers and bacteria may have synergistic effects in humans.^3^, ^10^, ^11^ There is an urgent need for studies to better understand the cellular processes involved in these beneficial immune effects of dietary fibers and/or beneficial bacteria,^12^ as it may lead to design of more effective strategies to prevent and/or manage inflammatory diseases.

Classically, the beneficial effects of food ingredients have been attributed to effects on gut microbiota and stimulation of its beneficial fermentation products such as short‐chain fatty acids (SCFAs).^13^ However, evidence is accumulating that immune active food ingredients such as dietary fibers and bacteria can also directly interact with the intestinal immune system.^14^, ^15^, ^16^, ^17^ For example, we have shown that inulin type fructans (ITF) can stimulate Toll‐like receptors (TLRs) on intestinal epithelial cells (IECs)^18^, ^19^ and on immune cells resulting in NF‐kB/AP‐1 activation and modulation of cytokine release from immune cells.^17^ Similar immune effects via pattern recognition receptors have been reported for lactobacilli.^14^, ^16^, ^20^ As a consequence of these interactions with pattern recognition receptors, these food ingredients may be instrumental, for example, in the management of STM infection as this enteropathogen uses TLRs,^21^ especially TLR2, 4, and 5, to invade the host.^22^

An important intestinal immune cell type involved in sensing of beneficial food ingredients are dendritic cells (DCs). DCs are one of the first immune cell types to come into contact with food compounds in the gut lumen.^23^, ^24^ Their role is to sense and phagocyte antigens and subsequently trigger adequate immune responses in other cells, such as T‐cells.^23^ In the intestine, DCs are located on strategic immune signaling locations such as in the lamina propria, mesenteric lymph nodes, and between IECs, where they continuously encounter luminal antigens.^25^ These luminal antigens may initiate different types of immune responses in DCs. The type of initiated response is determined by the composition of the antigen^26^ and (co)stimulation in the gut lumen of the innate immune receptors such as TLRs.^17^, ^27^ In DCs, the activation of specific pattern recognition receptors led to different cellular response which can regulate innate and adaptive immunity.^23^, ^24^

The DCs in the intestine experience cross talk with the IECs which are the first cells to encounter beneficial food components such as dietary fibers and lactobacilli.^25^ These IECs release, to be characterized, regulatory factors upon encounter with dietary fibers that attenuate inflammatory responses in DCs.^28^ This anti‐inflammatory effect is dietary fiber type dependent and may even change T‐cell responses in a dietary fiber specific way.^17^ Similar effects have also been shown with bacterial food components such as lactobacilli and are strain‐dependent.^29^, ^30^ However, possible direct synbiotic effects of dietary fibers and bacteria such as lactobacilli on immune cells have never been studied. Synbiotic effects are up to now mainly attributed to synergistic effects on microbiota.^31^, ^32^, ^33^

Here we studied possible direct synergistic effects of ITF and lactobacilli on DC immune responses. This was done by directly exposing DCs to the food ingredients in the presence and absence of media obtained from cultures of IECs that were exposed to the food ingredients for 20 h. This design allows us to conclude whether factors released by IECs during exposure to long‐chain inulin type fructans (lcITF) or a *Lactobacillus* strain modulate DC responses. We chose *Lactobacillus acidophilus* as study subject as this bacterium is known to modulate immune responses by modulating TLRs.^7^, ^20^, ^34^ Another reason to choose *L. acidophilus* was that we also studied the effects of ITF and lactobacilli alone and combined in an experimental setup where IECs were infected with STM. *L. acidophilus* is known to compete with STM for adhesion on IECs^35^, ^36^ and to induce direct immune effects which can support the DCs response toward the enteropathogen.^35^, ^37^ This allows us to conclude whether ITF and/or lactobacilli can be instrumental in the management of STM by directly interacting with intestinal cells.

## 2 Experimental Section

### 2.1 Ingredients and *S*. Typhimurium Culture

*L. acidophilus* strains W37 (LaW37; Winclove, Amsterdam, the Netherlands) was produced anaerobically at 37 °C, in modified MRS broth. Glycerol stocks were washed with PBS and resuspended in culture medium, brought to 37 °C, to reach 10^7^ CFU mL^−1^.

lcITF with DP10‐60 (Frutafit TEX!, Sensus, Cosun, Roosendaal, the Netherlands) was solubilized at 10 mg mL^−1^ in a culture medium at 37 °C and further diluted in medium to 5, 1, and 0.5 mg mL^−1^. The ITF was characterized by high‐performance anion exchange chromatography coupled with pulsed electrochemical detection, which was performed on an ICS5000 system (Thermo Fisher Scientific, Waltham, MA, USA), equipped with a Dionex CarboPac PA‐1 column (2 × 250 mm) in combination with a CarboPac PA‐1 guard column (2 × 50 mm; Figure S1, Supporting Information). The solution was 0.2 μm filtered to eliminate possible bacterial contaminations. Endotoxin concentrations in the filtered lcITF solutions were measured using the limulus amoebocyte lysate (LAL) that was carried out according to the manufacturer's protocol from Pierce LAL Chromogenic Endotoxin Quantitation Kit, Thermo Scientific (Pierce Biotechnology, Rockford, USA). Concentrations of endotoxins in lcITF filtered solutions fell below 0.3 × 10^−3^ endotoxin units per microgram (0.002 ng mL^−1^), which is too low to influence the results of the present study.

*S*. Typhimurium DT12 was provided by Trouw Nutrition (Boxmeer, the Netherlands). *S*. Typhimurium was grown in brain heart infusion (BHI) medium until stationary phase, was washed in PBS, and diluted so that the final concentration was 7.5 × 10^5^ CFU per transwell (tw).

### 2.2 HEK‐Blue SEAP Reporter Cell Assays

The human acute monocytic leukemia reporter cell line (THP‐1; InvivoGen, Toulouse, France) expresses endogenously, as previously described,^18^, ^38^ TLRs and an inserted construct for secreted embryonic alkaline phosphatase (SEAP) coupled to the NF‐κB and the AP‐1 transcription factor responsive promoter. This cell line also carries an extra insert for MD2 and CD14 that boosts TLR signaling. Furthermore, we used seven different human embryonic kidney (HEK)‐Blue reporter cell lines expressing one of human TLR2, 3, 4, 5, 7, 8, and 9 (InvivoGen, Toulouse, France). All seven cell lines also carried the inserted construct SEAP coupled to NF‐κB/AP‐1 promotor. Upon activation by their respective agonists, NF‐κB is transferred to the nucleus, the SEAP gene is expressed and can be measured in the supernatant using QuantiBlue reagent (InvivoGen, Toulouse, France).

THP‐1 cells were kept at a concentration of 5 × 10^5^ cells mL^−1^ and cultured in RPMI1640 supplemented with 10% heat‐inactivated fetal calf serum (hiFCS), 1.5 g L^−1^ NaHCO3 (Boom B.V. Meppel, the Netherlands), 2 mM l‐glutamine, 4.5 g L^−1^ glucose, 10 mM HEPES, 1 mM sodium pyruvate, and 50 U mL^−1^ penicillin per 50 μg mL^−1^ streptomycin, all purchased from Sigma Aldrich Chemie B.V. (Zwijndrecht, the Netherlands) and 100 μg mL^−1^ Normocin (InvivoGen). HEK‐Blue cells were maintained in Dulbecco's modified eagle's medium (DMEM) culture media (Lonza, Basel, Switzerland) with 10% hiFCS, 2 mM l‐glutamine, 4.5 g L^−1^ glucose, 50 U mL^−1^ penicillin per 50 μg mL^−1^ streptomycin, all from Sigma‐Aldrich and 100 μg mL^−1^ Normocin (InvivoGen). The culture medium was supplemented with selected antibiotics to maintain the stable expression of the PRR genes (see **Table** 1). HEK‐Blue cells were grown to approximately 80% confluence and were passaged three times in their respective selection media prior to any experiment, all according to manufacturer's instructions.

**Table 1:** Culturing specificities and agonists used in the HEK‐Blue reporter assays

HEK‐Blue cell line overexpressing	Selection antibiotics	Cell density [cells per mL]	Positive control (agonist)
TLR2	HEK‐Blue (1 μL mL^−1^)	2.8 × 10^5^	Heat‐killed *Listeria monocytogenes* (HKLM, 10^8^ cells mL^−1^)
TLR3	Blasticidin (30 μg mL^−1^) Zeocin (100 μg mL^−1^)	2.8 × 10^5^	Poly (I:C) low molecular weight (LMW, 1 μg mL^−1^)
TLR4	HEK‐Blue (1 μL mL^−1^)	1.4 × 10^5^	*Escherichia coli* K12 lipopolysaccharide‐HEK ultrapure (LPS, 0.1 μg mL^−1^)
TLR5	Blasticidin (30 μg mL^−1^) Zeocin (100 μg mL^−1^)	1.4 × 10^5^	Recombinant flagellin isolated from *Salmonella* Typhimurium (RecFLA‐ST, 0.1 μg mL^−1^)
TLR7	Blasticidin (10 μg mL^−1^) Zeocin (100 μg mL^−1^)	2.2 × 10^5^	9‐Benzyl‐8 hydroxyadenine derivative (CL264, 5 μg mL^−1^)
TLR8	Blasticidin (30 μg mL^−1^) Zeocin (100 μg mL^−1^)	2.2 × 10^5^	20‐Mer phosphorothioate single stranded RNA is complexed with the transfection reagent LyoVec (ssRNA40/LyoVec, 2 μg mL^−1^)
TLR9	Blasticidin (10 μg mL^−1^) Zeocin (100 μg mL^−1^)	4.5 × 10^5^	Type B CpG oligonucleotide (ODN2006, 10 μM)

### 2.3 Stimulation of Reporter Cells with LaW37, lcITF, and Their Combination

The HEK‐Blue reporter cell lines were seeded according to Table 1 in 200 μL per well in a 96 wells flat bottom culture plate and cultured overnight. The following day, medium was refreshed with 100 μL medium containing lcITF at 0.5, 1, 5, or 10 mg mL^−1^, LaW37 at 10^4^, 10^5^, or 10^6^ CFU per well, in combination, in the following ratio: 10^6^ CFU per well per 10 mg mL^−1^; 10^6^ CFU per well per 5 mg mL^−1^; 10^5^ CFU per well per 10 mg mL^−1^; 10^5^ CFU per well per 5 mg mL^−1^, or with the relevant agonist (Table 1), and the medium was used as negative control. For the inhibition assays, 10 μL of the corresponding agonist (Table 1) was added together with LaW37 and/or lcITF. THP‐1 cells were incubated with and without the addition of 50 μM Pepinh‐MyD88 (InvivoGen).

After 24 h of incubation at 37 °C 5% CO_2_, the supernatant of the cells was diluted at 1:4 with QuantiBlue solution. After 1 h incubation, a colorimetric measurement was performed at 650 nm on a Bio‐Rad Benchmark Plus microplate spectrophotometer reader (Bio‐Rad Laboratories B.V, Veenendaal, the Netherlands) using Bio‐Rad Microplate Manager 5.2.1 software. This color change was presented as fold‐change of NF‐κB activation. The assay was performed at least five times and each condition was performed in triplicates.

### 2.4 Caco‐2 Cell Culture and *S*. Typhimurium Challenge

ATCC derived Caco‐2 cells (HTB‐37, 2012) were cultured in DMEM (Gibco‐Invitrogen, Bleiswijk, the Netherlands) with 4.5 g L^−1^ glucose, 0.58 g L^−1^ glutamine, no pyruvate, and supplemented with 10% hiFCS (Hyclone Perbio, Etten‐Leur, the Netherlands). Cells were used within passage numbers 30 and 60, and 330 000 were seeded on ThinCert transwells with 33.6 mm^2^ membranes and 3 μm pores in 24‐well suspension culture plates. Cells were grown for 21 days at 5% CO_2_ and 37 °C. Apical (150 μL) and basolateral (700 μL) medium were replaced three times per week and on the day prior to the experiment.

LaW37 and lcITF were prepared within an hour prior to the experiment. Medium was then refreshed with or without the ingredient on the apical side. Each condition was tested in triplicate, and experiments were repeated six times, on different days. After 20 h incubation, the Caco‐2 cells were challenged with STM for 45 min after which the cells were washed in PBS apically and basolaterally, and subsequently refreshed with medium containing 100 μg mL^−1^ gentamicin (DMEM‐genta) on both sides. Basolateral medium of pooled replicates was collected after 4 h and the supernatant was stored at −80 °C for further experiments.

### 2.5 DCs Stimulation

**Figure 1:**
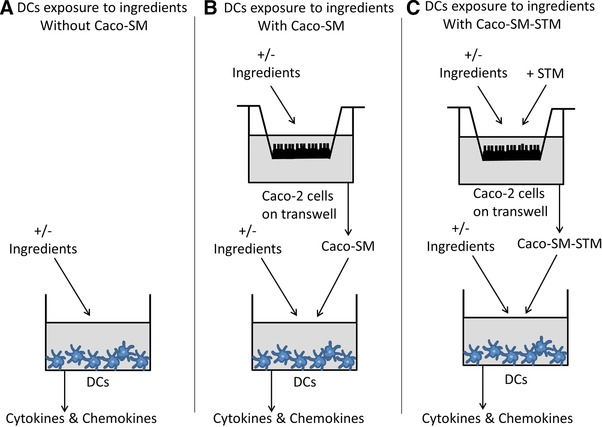
Experimental design for the DCs stimulation with long‐chain inulin type fructans (lcITF), *Lactobacillus acidophilus* W37 (LaW37), and lcITF/LaW37, in the A) absence and B) presence of Caco‐SM, C) with and without STM challenge. Effects of lcITF, LaW37, and lcITF/LaW37 on dendritic cells (DCs) directly exposed to these ingredients for 20 h (A) was compared to a cross‐talk situation where DCs are also exposed to the spent medium collected from Caco‐2 cells (Caco‐SM). For this purpose, Caco‐2 cells were cultured on transwells for 21 days in a separate plate and were incubated for 20 h with lcITF, LaW37, or lcITF/LaW37. The Caco‐SM collected was then transferred to the DCs that were separately cultured (B). In this setting, DCs are exposed concomitantly to this Caco‐SM and the corresponding, freshly prepared, ingredient. At last, Caco‐2 cells were challenged for 45 min to *Salmonella* Typhimurium (STM) and the same experiment was repeated (C).

To evaluate the direct immune effects of lcITF and/or LaW37 on intestinal cells, we used dendritic cells (DCs) and Caco‐2 epithelial cells. DCs were purchased from MatTek (MatTek Corporation, Ashland, MA, USA) and were generated from CD34+ progenitor cells harvested from umbilical cord blood which express HLA‐DR, CD83, and CD86, phenotypic maturation markers.^17^ The DCs resemble features of DCs found the in the gastrointestinal tract, such as presenting antigens to T‐cells, and have therefore been previously used in in vitro systems mimicking the gastrointestinal track.^14^, ^15^, ^16^, ^17^ We compared exposure of DCs to the ingredients with and without addition of Caco‐2 spent medium (Caco‐SM). The design for this series of experiments is described in **Figure** 1. These experiments were performed six times, as previously described,^17^, ^18^, ^39^ on different days.

DCs stimulation experiments were conducted with two different settings. First, DCs seeded onto a 96‐well plate (40 000 DCs per well) were incubated with 5 mg mL^−1^ of lcITF and/or 10^7^ CFU mL^−1^ of LaW37. After 20 h, DCs spent medium of triplicates were pooled and stored at −80 °C until further analysis (Figure 1A).

Next, we performed the same assay to which we added Caco‐SM. This Caco‐SM was collected from fully differentiated Caco‐2 cells grown on transwells as previously described and exposed to the same ingredient for 20 h. In this experiment, the DCs are exposed concomitantly to both the ingredients and the Caco‐SM collected after Caco‐2 cells were themselves exposed to that same ingredient in a 1:10 ratio. After DCs were stimulated for 20 h, the spent medium of triplicates were pooled and stored at −80 °C until further analysis (Figure 1B).

Furthermore, this experiment was repeated after exposure of Caco‐2 to STM. In this experiment, Caco‐2 cells pre‐incubated for 20 h with ingredients, were challenged with STM for 45 min as described in Section 2.4. The spent medium collected in the basolateral compartment is then referred to as Caco‐SM‐STM. Afterward, we proceed as in the previous experiment and stimulated DCs for 20 h with a simultaneous exposure to Caco‐SM‐STM and the corresponding ingredient. Spent medium of DCs collected from triplicates were pooled and stored at −80 °C until analysis (Figure 1C).

### 2.6 Luminex Analysis of Cytokines and Chemokines from DCs

The kit Magnetic Luminex premixed cytokine assay (R&D Systems Inc., Minneapolis, USA) was customized to simultaneously measure the following molecules in spent medium from DCs: IL‐12/23 p40, IL‐1ra, IL‐1β, IL‐6, MCP‐1/CCL2, CCL3/MIP‐1α, CCL‐5/RANTES, IFN‐γ, IL‐10, and TNF‐α.

Luminex assays were performed according to the manufacturer's instructions. Briefly, a concentration series of cytokine standards were prepared for the appropriate concentration range. The undiluted microparticle cocktail specific for DCs was added to each well (50 μL per well), washed, and standards, negative controls, and samples were all incubated overnight at 4 °C shaking (duplos, 50 μL per well). After incubation, the plate was washed three times, a biotin antibody cocktail was added to each well (50 μL per well) and the plate was further incubated while shaking for 1 h at room temperature (RT). The plate was washed three times and streptavidin–phycoerythrin was added to each well (50 μL per well). After 30 min incubation shaking at RT, the plate was washed three times and the microparticles were resuspended in 100 μL of wash buffer. Fluorescence was then measured within 90 min using a Luminex analyzer MAGPIX and xPONENT 4.2 for MAGPIX software (Luminex Corporation, http://‘s-Hertogenbosch, the Netherlands).

### 2.7 ELISA Analysis of IL‐8 Produced by DCs

IL‐8 was measured in spent medium from DCs using a human CXCL8/IL‐8 DuoSet ELISA ELISA kit (R&D systems), performed according to manufacturer's instruction. In short, 96‐well plates (R&D systems) were coated with capture antibodies at a concentration of 4 μg mL^−1^ overnight at RT after which the plates were washed with a filtered block buffer containing 1% BSA (Sigma Aldrich). Samples were diluted 1:4 and incubated for 2 h after which the detection antibody was added at a concentration of 20 ng mL^−1^ and incubated for 2 h. Reaction with 40‐fold diluted streptavidin‐horse radish peroxidase occurred afterward for 20 min and was followed by 20 min reaction with 3,3′,5,5′‐tetramethylbenzidine (Sigma Aldrich) that was stopped with carboxylic acid (BioLegend Inc., San Diego, CA, USA). Plates were thoroughly washed three times with wash buffer containing 0.05% TWEEN. Optical density was immediately estimated at 540 nm with subtraction of 450 nm background using Bio‐Rad Benchmark Plus microplate spectrophotometer reader. Data were processed using GraphPad Prism version 7.0a (GraphPad Software, Inc., La Jolla, USA).

### 2.8 Statistical Analysis

THP‐1 and HEK‐Blue cells TLRs data were normalized compared to medium control so that medium equals one, and activation value can be expressed as fold‐change induction of NF‐κB/AP‐1 compared to medium. The data were not normally distributed as confirmed by the Kolmogorov–Smirnov test. Statistical differences were analyzed using Kruskal–Wallis followed by Dunn's post hoc test. Cytokines data were normally distributed as confirmed by the Kolmogorov–Smirnov test and ANOVA was applied followed by LSD test. *p*‐values <0.05 were considered to be statistically significant and *p* < 0.1 was a trend. Cytokines data are expressed as average (pg mL^−1^) ± SEM. All data were analyzed with GraphPad Prism.

## 3 Results

The aim of this study was to determine whether direct immune effects of dietary fibers such as lcITF and a lactic acid bacterium like LaW37 are synergistic and modulates mucosal immunity during infection with an enteropathogen by directly interacting with immune receptors. We first established possible activation of crucial pathogens recognition receptors, the TLRs. Next, we investigated whether these effects were associated with immune cell signaling in DCs, and whether this response was modulated by factors released by IECs after Caco‐2 were challenged or not with STM. At last, we evaluated the effects of the ingredients on DCs with and without STM challenge.

### 3.1 Immune Receptors Are Specifically Reacting to LaW37, lcITF, and Their Combination Has Synergistic Effects

**Figure 2:**
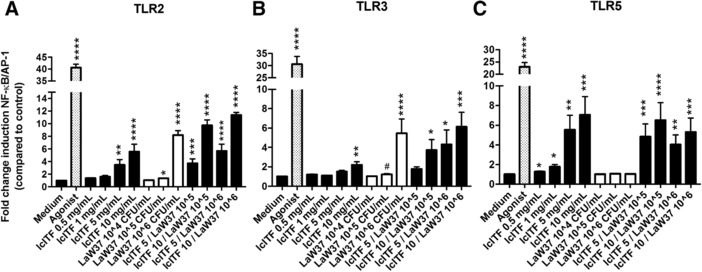
NF‐ΚB/AP‐1 activation in HEK‐Blue reporter cell lines expressing A) TLR2, B) 3, or C) 5 by lcITF, LaW37, and lcITF/LaW37. Agonists used were, respectively, heat‐killed *Lysteria monocytogenes*, Poly (I:C) low molecular weight, and *Salmonella* Typhimurium flagellin. Long‐chain inulin type fructans (lcITF) was tested at 0.5, 1, 5, and 10 mg mL^−1^, and *Lactobacillus acidophilus* W37 (LaW37) was tested at 10^4^, 10^5^, and 10^6^ CFU mL^−1^; *n *= 5 performed in triplicates. Statistical significances compared to medium were assessed by Kruskal–Wallis with Dunn's test and *p < 0.05, **p < 0.01, ***p < 0.001, ****p < 0.0001, and a trend is #*p* < 0.1.

As both lcITF and lactobacilli have been shown to signal on immune cells via TLRs,^18^, ^20^ we first investigated possible synergistic effects on reporter cell‐lines expressing TLR2, 3, 4, 5, 7, 8, and/or 9. Activation by lcITF was observed in TLR2, 3, and 5 expressing cell lines. Therefore, only these results are shown in **Figure** 2. LcITF had a strong dose‐dependent activating effect on TLR2 (Figure 2A) up to 3.5‐fold (*p* < 0.0001) at 5 mg mL^−1^. Activation of TLR3 was reaching 1.5‐fold (*p *= 0. 001) at 5 mg mL^−1^ (Figure 2B). LcITF induced activation of TLR5 was dose‐dependent (Figure 2C) with a 5.6‐fold increase (*p* < 0.0001) at 5 mg mL^−1^.

LaW37 statistically significantly activated TLR2 in a dose‐dependent way inducing a 1.4‐fold increase at 10^6^ CFU mL^−1^ (*p *= 0.003) and an 8.2‐fold increase (*p *< 0.0001) at 10^7^ CFU mL^−1^ (Figure 2A). A trend toward increase of TLR3 was observed at 10^6^ CFU mL^−1^ (1.2‐fold; *p *= 0.07) and a statistically significant increase reached 5.5‐fold at 10^7^ CFU mL^−1^ (*p *= 0.0002; Figure 2B). The combination lcITF/LaW37 had synergistic effects in TLRs.

The combination activated TLR2 in a dose‐dependent manner with a 5.7‐fold increase at 5 mg mL^−1^ per LaW37 10^6^ CFU mL^−1^ (*p *= 0.003) and 11.4‐fold increase at lcITF 10 mg mL^−1^ per LaW37 10^6^ CFU mL^−1^ (*p* < 0.0001; Figure 2A). It also activated TLR3 in a synergistic way. LcITF 10 mg mL^−1^ / LaW37 10^6^ CFU mL^−1^ led to 6.1‐fold increase (*p* < 0.0001; Figure 2B). LcITF/LaW37 statistically significantly activated TLR5 in a dose‐dependent manner (Figure 2C), this however did not differ from what was observed for lcITF alone.

### 3.2 LaW37 Stimulates a Pro‐Inflammatory Phenotype in DCs While lcITF/LaW37 Attenuates the Response in DCs

After confirming immune signaling via TLRs and synergistic effects of lcITF and LaW37, we studied the effect of lcITF and LaW37 on DCs to determine the final effects on immune signaling (Figure 1A). We investigated the effects of lcITF, LaW37, and their combination on human IL‐12/23 p40, IL‐1ra, IL‐1β, IL‐6, IL‐8, MCP‐1/CCL2, CCL3/MIP‐1α, CCL‐5/RANTES, IFN‐γ, IL‐10, and TNF‐α production (**Table** 2).

**Table 2:** Effect of direct exposure of long‐chain inulin type fructans (lcITF), *Lactobacillus acidophilus* W37 (LaW37), and lcITF/LaW37 on DC cytokine responses. Effects of 5 mg mL^−1^ lcITF, 10^7^ CFU mL^−1^ LaW37, and lcITF/LaW37 on IL‐1ra, IL‐6, IL‐8, MCP‐1/CCL2, and TNF‐α productions were measured by Luminex in a medium of DCs directly exposed to ingredients for 20 h. Data are averages with SEM values of six repetitions, with triplicates. Statistical significances of differences compared to unstimulated DCs were tested in GraphPad Prism ANOVA with LSD test and ***p < 0.001, *p < 0.05, and #*p* < 0.1

DCs exposed to	Unstimulated DCs		lcITF		LaW37		lcITF/LaW37	
[pg mL^−1^]	Average	SEM	Average	SEM	Average	SEM	Average	SEM
CCL‐2/MCP‐1	64,1	8,6	43,9	9,3	154,9*	33,5	129^#^	32,5
IL‐1ra	1587	107	1228	147,3	2244	283,2	3270	1074
IL‐6	2,1	0,6	2	0,5	22,3***	6,8	9,7	2,9
TNF‐α	3,5	0,7	3,3	0,8	27,4***	5,8	16,1*	3
IL‐8	565,8	167,7	912,8	247,7	674,6	157,9	683,1	113,2

The production of IL‐1β, CCL3/MIP‐1α, CCL‐5/RANTES, IFN‐γ, IL‐12.23p40, and IL‐10 was below detection levels at all occasions. There was no statistically significant effect of lcITF on DCs. This was different for LaW37. LaW37 statistically significantly increased CCL‐2/MCP‐1 (*p *= 0.022), IL‐6 (*p *= 0.001), and TNF‐α (*p *= 0.001) compared to unstimulated DCs (Table 2). Finally, the combination lcITF/LaW37 statistically significantly increased TNF‐α (*p *= 0.026) and tended to increase CCL‐2/MCP‐1 (*p *= 0.084; Table 2). In general, the effects of the combination lcITF/LaW37 were milder than those observed for LaW37 as pro‐inflammatory IL‐6 (*p *= 0.019) and TNF‐α (*p *= 0.027) were statistically significantly highly enhanced by LaW37 than by lcITF/LaW37.

### 3.3 DC Responses to lcITF and/or LaW37 Are Modulated by Intestinal Epithelial Cells

As it is well known that IECs derived factors are essential for modulating responses of DCs when exposed to dietary fibers,^17^, ^25^ we repeated the experiment to investigate whether IECs spent medium (Caco‐SM), collected after 20 h exposure to lcITF, LaW37, or lcITF/LaW37, can modulate the DCs response (Figure 1B). This was done by incubating DCs in a 1:10 ratio of Caco‐SM for 20 h. We abandoned cocultures of IECs and DCs and preferred to include Caco‐SM instead of DCs as this set up allows us to exclusively measure the DCs response. We investigated the effects of lcITF, LaW37, or lcITF/LaW37 on human IL‐12/23 p40, IL‐1ra, IL‐1β, IL‐6, MCP‐1/CCL2, CCL3/MIP‐1α, CCL‐5/RANTES, IFN‐γ, IL‐10, and TNF‐α production in spent medium from DCs as presented in **Table** 3.

**Table 3:** Effect of direct exposure of long‐chain inulin type fructans (lcITF), *Lactobacillus acidophilus* W37 (LaW37), and lcITF/LaW37 on DC cytokine responses in the presence of IEC media. The effects of 5 mg mL^−1^ lcITF, 10^7^ CFU mL^−1^ LaW37, and lcITF/LaW37 on IL‐1ra, IL‐6, IL‐8, MCP‐1/CCL2, and TNF‐α productions by DCs after 20 h exposure to the ingredients combined with Caco‐2 intestinal epithelial cells (IECs) medium (Caco‐SM) were measured by Luminex. Caco‐2 cells were incubated 20 h, beforehand, with lcITF, LaW37, or lcITF/LaW37. Data are averages with SEM values of six repetitions, with triplicates. Statistical significance of differences compared to DCs exposed to unstimulated Caco‐SM were tested in GraphPad Prism ANOVA with LSD test and ****p < 0.0001, **p < 0.01, *p < 0.05, and #*p* < 0.1

DCs exposed to	Caco‐SM		lcITF + Caco‐SM		LaW37 + Caco‐SM		lcITF/LaW37 + Caco‐SM	
[pg mL^−1^]	Average	SEM	Average	SEM	Average	SEM	Average	SEM
CCL‐2/MCP‐1	84,4	12,7	68	9	124,4	16,4	119,2	14,2
IL‐1ra	1575	124	1288	118,3	1982^#^	258,2	1885	180,8
IL‐6	5,8	1,8	6,4	1,9	13,7	5,4	21,4*	5,5
TNF‐α	10,5	2,3	8	2	19,6^#^	2,9	23,6**	4,2
IL‐8	556,3	67,9	475,4	51,0	1117****	85	1367****	106,9

First, we assessed the effect of the Caco‐SM itself on DCs and compared cytokine levels to that of unstimulated DCs. As shown in Table 3, there was no statistically significant difference for the tested cytokines and chemokines.

Next, we exposed DCs to both the ingredients and Caco‐SM after Caco‐2 cells were themselves exposed for 20 h to the same ingredients. The production of IL‐1β, CCL3/MIP‐1α, CCL‐5/RANTES, IFN‐γ, IL‐12.23p40, and IL‐10 was below detection levels at all occasions. There was no statistically significant difference for lcITF. This was different for LaW37. LaW37 statistically significantly increased the pro‐inflammatory chemokine IL‐8 (*p* < 0.0001) and tended to increase TNF‐α (*p *= 0.064) and IL‐1ra (*p *= 0.105) compared to DCs exposed to unstimulated Caco‐SM (Table 3). Finally, the combination lcITF/LaW37 statistically significantly increased IL‐8 (*p* < 0.0001), TNF‐α (*p *= 0.0035), and IL‐6 (*p *= 0.023) (Table 3). The combination lcITF/LaW37 had stronger effects on pro‐inflammatory cytokines than LaW37 alone and increased the production of the pleiotropic IL‐6 but not of the anti‐inflammatory IL‐1ra, which was the opposite for LaW37 alone. Moreover, synergistic effects by lcITF/LaW37 were observed on IL‐8 production as IL‐8 tended to be highly enhanced by lcITF/LaW37 than by LaW37 (*p *= 0.055) and lcITF (*p* < 0.0001) alone.

### 3.4 STM Challenge of Caco‐2 Cells Lowers TNF‐α Responses in DCs Response

Next, we applied an STM challenge to Caco‐2 cells to study effect of lcITF, LaW37, or lcITF/LaW37 on STM induced inflammatory responses in IECs. STM is an enteropathogen capable of escaping the immune recognition, invading epithelial cells, and being internalized by DCs.^40^, ^41^ We repeated the above experiment on DCs but added a 45 min STM challenge of Caco‐2 cells after they were pre‐incubated for 20 h with lcITF, LaW37, or lcITF/LaW37 (Figure 1C). The medium collected from the basolateral side of the Caco‐2 culture exposed to both ingredients and STM is further referred to as Caco‐SM‐STM.

As control, we first analyzed the cytokine levels produced by DCs after exposure to Caco‐SM‐STM in the absence of ingredients by measuring production of human IL‐12/23p40, IL‐1ra, IL‐1β, IL‐6, MCP‐1/CCL2, CCL3/MIP‐1α, CCL‐5/RANTES, IFN‐γ, IL‐10, and TNF‐α after 20 h incubation. This was compared to responses of DCs that were exposed to Caco‐SM without *S*. Typhimurium exposure. When DCs were exposed to Caco‐SM‐STM, the production of IL‐1β, CCL3/MIP‐1α, CCL‐5/RANTES, IFN‐γ, IL‐12/23p40, and IL‐10 was below detection levels at all occasions. As presented in **Table** 4, challenge of Caco‐2 with STM did not induce any statistically significant change in CCL‐2/MCP‐1, IL‐1ra, IL‐6, or IL‐8. However, TNF‐α was reduced from 10 pg mL^−1^ in medium control to over 4 pg mL^−1^ (*p* < 0.0001) in DCs exposed to Caco‐SM‐STM compared to DCs exposed to Caco‐SM control (Table 4).

**Table 4:** Effect of direct exposure of long‐chain inulin type fructans (lcITF), *Lactobacillus acidophilus* W37 (LaW37), and lcITF/LaW37 on DC cytokine responses in the presence of IEC media exposed to *Salmonella* Typhimurium (STM) DT12 in combination with the ingredients. The effects of 5 mg/mL lcITF, 10^7^ CFU mL^−1^ LaW37, and lcITF/LaW37 on IL‐1ra, IL‐6, IL‐8, MCP‐1/CCL2, and TNF‐α production by DCs after 20 h exposure to the ingredients combined with Caco‐2 intestinal epithelial cells (IECs) medium (Caco‐SM‐STM) were measured by Luminex. Caco‐2 cells were incubated 20 h, beforehand, with lcITF, LaW37, or lcITF/LaW37 and challenged with STM for 45 min. Data are averages with SEM values of six repetitions, with triplicates. Statistical significance of differences compared to DCs exposed to Caco‐SM‐STM, without ingredients, were tested in GraphPad Prism ANOVA with LSD test and ****p < 0.0001, ***p < 0.001, **p < 0.01, and *p < 0.05

DCs exposed to	Caco‐SM‐STM		lcITF + Caco‐SM‐STM		LaW37 + Caco‐SM‐STM		lcITF/LaW37 + Caco‐SM‐STM	
[pg mL^−1^]	Average	SEM	Average	SEM	Average	SEM	Average	SEM
CCL‐2/MCP‐1	70,6	17,4	93,8	13,8	137,3**	17,9	124,6*	14,9
IL‐1ra	1502	195	1488	81,4	1825	182,9	1794	175,2
IL‐6	1,8	0,4	6,5	3	22,7**	6,6	27,8***	7,9
TNF‐α	7,3	2,4	6,7	1,2	27***	4,3	26,6***	3,9
IL‐8	517,7	87,1	577,8	70,7	1258****	140,7	1422****	116,1

Next, we analyzed the effects on DCs of lcITF, LaW37 and lcITF/LaW37 on STM challenged IECs. We therefore repeated this experiment with pre‐incubation of Caco‐2 cells to lcITF, LaW37, or lcITF/LaW37 for 20 h prior to the 45 min STM challenge. This Caco‐SM‐STM was then exposed to DCs cultured separately in a 1:10 ratio. DCs were concomitantly exposed to Caco‐SM‐STM and freshly prepared lcITF, LaW37, or lcITF/LaW37. The DCs response was analyzed by measuring the production of human IL‐12/23 p40, IL‐1ra, IL‐1β, IL‐6, MCP‐1/CCL2, CCL3/MIP‐1α, CCL‐5/RANTES, IFN‐γ, IL‐10, and TNF‐α after 20 h incubation. Production of IL‐1β, CCL3/MIP‐1α, CCL‐5/RANTES, IFN‐γ, IL‐12/23p40, and IL‐10 was below detection level at all occasions. As presented in Table 4, lcITF had no statistically significant effect on any of the detectable cytokines and chemokines. This was different for LaW37. LaW37 increased all detectable cytokines and this was statistically significant for CCL‐2/MCP‐1 (*p *= 0.004), IL‐6 (*p *= 0.007), IL‐8 (*p* < 0.0001), and TNF‐α (*p *= 0.0001) but not for IL‐1ra. The same was observed for the combination lcITF/LaW37.

## 4 Discussion

Our study shows for the first time, to the best of our knowledge, that dietary fibers and bacterial supplementation can synergistically influence immunity by directly interacting with immune cell receptors, and eventually modulate the DC responses. This might be another mechanism for synbiotic effects than the microbiota driven and indirect modulation of immunity so far observed in other studies.^31^, ^32^, ^33^ Also, our study shows that DCs directly exposed to LaW37 or lcITF/LaW37 react differently than the DCs that are also exposed to IECs media. In the first case, direct effects of LaW37 were dampened by the addition of lcITF, while enhancing effects on IL‐6 and IL‐8 were observed in the presence of IECs.

The selected dietary fiber lcITF showed direct stimulatory effects on TLR2, 3, and 5. Effects on TLR2 and 5 were dose‐dependent as reported before.^18^ Despite this observation, direct stimulation of DCs did not lead to any change in cytokine and chemokine production. This can be explained by differential and simultaneous stimulation of pattern recognition receptors leading to activation of several downstream pathways, bringing regulatory and pro‐inflammatory responses into balance, as reported before.^17^, ^38^ Another factor could be due to varying expression of TLRs as different DC population may have different phenotypic reactions to TLR activation.^42^ Despite the absence of immune stimulation of DCs by lcITF, we decided to test effects of lcITF in the context of an STM infection of IECs as lcITF stimulates, as shown in our study, TLR5. The main ligand for TLR5 is flagellin, a pathogenic molecule expressed by STM. Unfortunately, during the STM infection, lcITF did not enhance the DCs responses.

Stimulatory effects of the *Lactobacillus* strain LaW37 were observed on TLR2 and 3. This corroborates the observation of others.^7^ TLR2 activation should be explained by the production of lactic acid and presence of lipoteichoic acid on LaW37, which are both specific ligands of TLR2.^20^, ^34^ Although change of pH and production of lactic acid were not quantified, acidification of the medium was not observed during stimulation of the cells suggesting low metabolic activity of LaW37. Notably, however, the potential of LaW37 to activate TLRs is specific as not all lactobacilli were reported to activate TLRs, for example, *Lactobacillus paracasei*.^14^ The effects of LaW37 on direct immune stimulation were confirmed by exposing DCs to LaW37 which resulted in enhanced production of the chemokine CCL‐2/MCP‐1 and the pleiotropic IL‐6. It also tended to increase the pro‐inflammatory cytokine TNF‐α which is a more general feature of lactic acid producing bacteria.^43^ Moreover, in this same study, effects of *L. acidophilus* were shown to be specific and particularly efficient at increasing expression of HLA‐DR, CD40, and CD86 DCs surface maturation markers^43^ which are also present on the DCs used in our study and could explain the pro‐inflammatory effects.

IEC media modulated responses of DCs exposed to LaW37. Production of the pro‐inflammatory cytokines and chemokines IL‐8 and TNF‐α was increased in the presence of IEC media after Caco‐2 cells were exposed to LaW37. Moreover, it implies that bacteria such as *L. acidophilus* may have other effects on IECs than dietary fibers as in a previous study we showed that dietary fibers exclusively attenuate immune responses in DCs.^17^ Our observation is in line, however, with studies of others demonstrating the capability of lactobacilli to induce pro‐inflammatory responses in vivo^6^ and in DCs.^44^ Notably, the induction of IL‐8 in DCs was not observed unless Caco‐SM was present suggesting that the induction of IL‐8 production by DCs is likely to be a consequence of IEC derived factors induced by lactobacilli.

The combination of lcITF and LaW37 had synergistic effects on TLR2 and 3 activations which suggest that the mechanisms behind synbiotic effects might also include synergistic effects on immune receptors such as TLRs. As effects on inflammatory responses of combined stimulation of pattern recognition receptors are difficult to predict,^15^ we also performed studies of synbiotic effect on immune response of DCs. Direct exposure to lcITF/LaW37 led to increased CCL‐2/MCP‐1 and TNF‐α. This increase was statistically significantly lower than for LaW37 alone. This indicates that lcITF/LaW37 together give different responses in DCs and thus have synergistic effects which can be attenuation of inflammatory responses and induction of more regulatory responses. Interestingly, this has already been shown on IECs, also with a combination of lcITF and *L. acidophilus*.^45^

Synergistic effects of lcITF/LaW37 were also observed when Caco‐SM was added to DCs. LcITF/LaW37 induced production of IL‐8 and TNF‐α. IL‐8 increase was higher for the combination than for LaW37 alone. Moreover, lcITF/LaW37 also induced IL‐6 production, which was unique to the combination. To study whether the observed synergistic effects can have any functional meaning, DCs were, next, exposed to IECs infected with STM. After STM challenge, lcITF/LaW37 increased CCL‐2/MCP‐1, TNF‐α, IL‐8 and IL‐6 production by DCs. There was no difference compared to LaW37. This is different than the results of Huang et al. where gene expression of IL‐8 and TNF‐α in IECs during STM challenge was decreased by *L. acidophilus* alone or combined with lcITF.^45^ Our experiments solely focused on DCs responses which were not directly exposed to STM, as we mimicked the in vivo situation where DCs are protected by the IECs. Therefore, during STM challenge, DCs in our experiments were not under a pro‐inflammatory setting unlike the IECs used in the Huang et al. study which might explain such differences. Another difference is that the combination used by Huang et al. had stronger effects than *L. acidophilus* alone^45^ which suggests that synbiotics can have a synergistic effect during STM infection, although no synergistic effect could be shown in our study. Moreover, in our study, LaW37 already strongly enhanced CCL‐2/MCP‐1, TNF‐α, IL‐8, and IL‐6 that it is likely that no significant effect of the modest cytokine enhancing dietary fiber lcITF can be measured.

In conclusion, we demonstrated that synbiotic effects of the dietary fiber lcITF and lactic bacteria LaW37 occurred by synergistically activating immune receptors. In general, LaW37 displayed pro‐inflammatory effects on DCs no matter the conditions which is likely due to its capacity to stimulate TLR2 and 3. The combination lcITF/LaW37 was found to synergistically activate specific TLRs and had unique effects on DCs cytokine profiles. However, during STM challenge, no added effects of the combination were observed which should probably be explained by the strong effects of LaW37 on the DCs cytokine response in the presence of STM infection. This demonstrates for the first time, to the best of our knowledge, that synbiotic effects of dietary fibers and bacteria are not limited to effects on gut microbiota but can also occur by synergistically directly stimulating IECs and/or immune cells. The final outcome of such a synergistic effect depends on the strength by which the individual ingredient modulates immune responses.

## Conflict of Interest

The authors declare no conflict of interest

## Supporting information

Supplementary Figure S1. Long‐chain inulin‐type fructan (lcITF; Frutafit® TEX!) HPAEC profile. Peaks represent fructose (F) and glucose (G) monomers, dimers and fructans oligomers present in the formulation of lcITF. GFn and Fn chains respectively terminated by a glucose or fructose molecule with n the number of fructose moieties in the chain.
