# Supplementary material for: Synbiotic Effects of the Dietary Fiber Long‐Chain Inulin and Probiotic Lactobacillus acidophilus W37 Can be Caused by Direct, Synergistic Stimulation of Immune Toll‐Like Receptors and Dendritic Cells
Source: Mol Nutr Food Res. 2018 Jul 18;62(15):1800251. doi: 10.1002/mnfr.201800251 (PMC6099370; doi:10.1002/mnfr.201800251)
Supplement: Supplementary file 1 — Supplementary Figure S1. Long‐chain inulin‐type fructan (lcITF; Frutafit® TEX!) HPAEC profile. Peaks represent fructose (F) and glucose (G) monomers, dimers and fructans oligomers present in the formulation of lcITF. GFn and Fn chains respectively terminated by a glucose or fructose molecule with n the number of fructose moieties in the chain. [file MNFR-62-na-s001.docx]

**Supporting Information**

**Figures**





GF25

GF20

GF15

GF10

GF5

GF

G

F

**Supplementary Figure S1. Long-chain inulin-type fructan (lcITF; Frutafit® TEX!) HPAEC profile.** Peaks represent fructose (F) and glucose (G) monomers, dimers and fructans oligomers present in the formulation of lcITF. GFn and Fn chains respectively terminated by a glucose or fructose molecule with n the number of fructose moieties in the chain.
